# Site Specific Genetic Incorporation of Azidophenylalanine in *Schizosaccharomyces pombe*

**DOI:** 10.1038/srep17196

**Published:** 2015-11-24

**Authors:** Nan Shao, N. Sadananda Singh, Susan E. Slade, Alexandra M. E. Jones, Mohan K. Balasubramanian

**Affiliations:** 1 Division of Biomedical Cell Biology, Warwick Medical School, University of Warwick, Coventry, UK CV4 7AL; 2 Department of Biological Sciences, National University of Singapore, Singapore 117543; 3 Temasek Life Sciences Laboratory, National University of Singapore, 1 Research Link, Singapore 117604; 4 School of Life Sciences, University of Warwick, Coventry, UK CV4 7AL; 5 Mechanobiology Institute, 5A Engineering Drive 1, National University of Singapore, Singapore 117411

## Abstract

The diversity of protein functions is impacted in significant part by the chemical properties of the twenty amino acids, which are used as building blocks for nearly all proteins. The ability to incorporate unnatural amino acids (UAA) into proteins in a site specific manner can vastly expand the repertoire of protein functions and also allows detailed analysis of protein function. In recent years UAAs have been incorporated in a site-specific manner into proteins in a number of organisms. In nearly all cases, the amber codon is used as a sense codon, and an orthogonal tRNA/aminoacyl-tRNA synthetase (RS) pair is used to generate amber suppressing tRNAs charged with the UAA. In this work, we have developed tools to incorporate the cross-linking amino acid azido-phenylalanine (AzF) through the use of bacterial tRNA^Tyr^ and a modified version of TyrRS, AzFRS, in *Schizosaccharomyces pombe*, which is an attractive model organism for the study of cell behavior and function. We have incorporated AzF into three different proteins. We show that the majority of AzF is modified to amino-phenyl alanine, but protein cross-linking was still observed. These studies set the stage for exploitation of this new technology for the analysis of *S. pombe* proteins.

Twenty canonical amino acids are used as building blocks to construct proteins in all organisms. The sequences of amino acids and their chemical properties give rise to the large array of activities encoded in proteins. Interestingly, some non-canonical amino acids, like pyrrolysine, selenocysteine, exist in living systems and are incorporated into proteins through endogenous translation systems^1^,^2^,^3^. Large arrays of unnatural amino acids have been developed through chemical approaches. Incorporation of these amino acids at precise locations in proteins should lead to novel ways of studying protein function as well as improved biotechnological applications.

Addition of new building blocks to the natural amino acids requires a unique codon which has not been assigned to any natural amino acid^4^. In addition, an orthogonal tRNA/aminoacyl-tRNA synthetase pair, which does not cross-react with any endogenous translation components has to be introduced into host cells to incorporate the unnatural amino acid(s) at specified locations^4^,^5^,^6^. Genetic code expansion and protein functional studies have been achieved in many organisms and was first described in *Escherichia coli*^7^,^8^. Now more than 80 unnatural amino acids with novel chemical, physical, and biological properties^8^ have been successfully incorporated into eukaryotic model organisms or cell lines in response to the amber codon TAG, including in *Saccharomyces cerevisiae*^9^, *Candida albicans*^10^, *Caenorhabditis elegans*^11^, mammalian somatic cells and stem cells^12^,^13^.

Fission yeast *Schizosaccharomyces pombe* is an attractive model for understanding molecular mechanisms governing cell growth, division, and polarity. *S. pombe* is amenable to experimental manipulation using methods of genetics, molecular biology, biochemistry, genomics, and high resolution imaging. To expand the repertoire of technologies that can be used in fission yeast to study protein function, we sought to develop experimental approaches to expand the fission yeast genetic code and thereby to facilitate genetic incorporation of unnatural amino acids at specified locations into fission yeast proteins.

In this study, we expanded the genetic code of *S. pombe*. We have engineered *S. pombe* cells with an orthogonal *E. coli* tyrosyl-tRNA synthetase (TyrRS)/*E. coli* tRNA^Tyr^ pair, enabling site specific incorporation of natural and unnatural amino acids through amber codon suppression. We show that the cross-linking nitrene generating amino acid azido-phenylalanine (AzF) can be introduced into three different proteins in fission yeast. We also show the ability of glutathione S-transferase containing AzF at position 52 (the dimerization site) to generate covalently linked multimers upon UV exposure. Our studies introduce a new and powerful approach to study function of fission yeast proteins.

## Results

### Expression and functionality of the orthogonal pair in *S. pombe*

Incorporation of the unnatural amino acids at a specified location within a protein of interest is achieved by the availability of 3 elements: 1. a codon that does not encode any natural amino acid and can encode the unnatural amino acid when present in the mRNA. 2. a tRNA that base-pairs with this codon and 3. an aminoacyl-tRNA synthetase that charges the tRNA with the unnatural amino acid^9^. It is also important that the tRNA and the tRNA synthetase are orthogonal. The amber codon (UAG) has been developed as a sense codon for the incorporation of several unnatural amino acids into proteins in recent years. It has been established that the tRNA for tyrosine (tRNA^Tyr^) from *Escherichia coli* or *Bacillus sterothermophillus* and the tyrosyl-tRNA synthetase (TyrRS) from *E. coli* function as an orthogonal pair in many eukaryotes^12^,^14^. Furthermore, mutant versions of *E. coli*/*B. stearothermophillus* (tRNA^Tyr^) with mutations in the anti-codon loop that base-pair with amber codon and aaRS that can charge these tRNAs with tyrosine or UAAs have been generated^9^. We therefore chose to use this orthogonal tRNA^Tyr^ and TyrRS pair for genetic code expansion studies in fission yeast.

**Figure 1:**
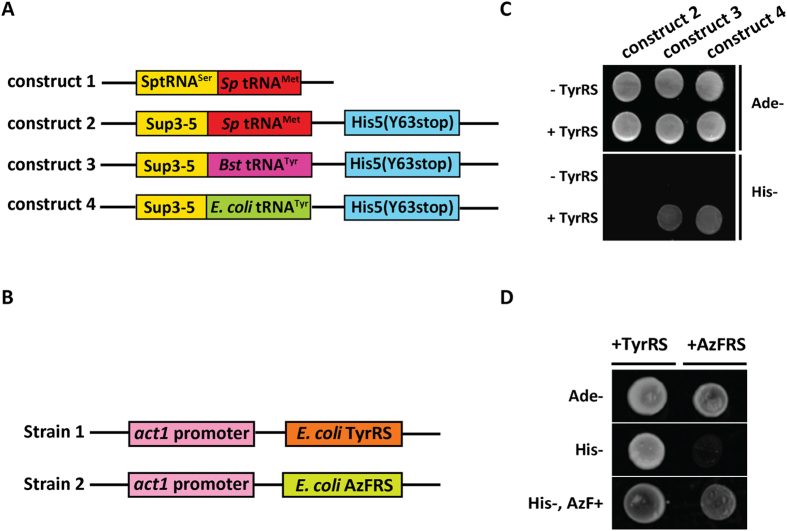
Suppression by orthogonal tRNA/aaRS pairs in S. pombe. (**A**) Schematic representation of the DNA constructs. Construct 1: expressing *S. pombe* methionine tRNA (*Sp* tRNA^Met^) and *S. pombe* serine tRNA (*Sp* tRNA^Ser^). Construct 2: expressing *S. pombe* methionine tRNA (*Sp* tRNA^Met^) and *sup3–5* (an anticodon mutant of *S. pombe* tRNA^ser^). Construct 3: expressing *B. stearothermophilus* tyrosine tRNA carrying CUA in the anti-codon loop (*Bst* tRNA^Tyr^) and *sup3–5*. Construct 4: expressing *E. coli* tyrosine tRNA carrying CUA in the anti-codon loop (*E. coli* tRNA^Tyr^) and *sup3–5*. Constructs 2–4 carry a selectable marker, His5p, which carries an amber stop codon at position 63. (**B**) Strain construction. Strain 1: *E. coli* TyrRS was integrated at the *leu1* locus and expressed under *act1* promoter. Strain 2: *E. coli* AzFRS was integrated at the *leu1* locus and expressed under *act1* promoter. (**C**) Spot assay of amber codon suppression in *S. pombe*. The numbers above the cell spot assay images represents the DNA constructs (schematized in Fig. 1A) that were introduced into the *ade6–704 his5Δ* strain. (**D**) Spot assay for the AzF incorporation into His5p. In each blot, the left colony is formed by the *S. pombe* strain expressing *E. coli* TyrRS and *E. coli* tRNA^Tyr^. The right colony is formed by the *S. pombe* strain expressing *E. coli* AzFRS and *E. coli* tRNA^Tyr^. Both strains are plated in the minimal medium lacking adenine (top), histidine (middle), and histidine supplemented with 1 mM AzF (bottom), respectively.

To expand the genetic code of *S. pombe*, we first designed a system to express tRNAs. It is known that an *S. pombe* tRNA^Ser^ is co-transcribed with tRNA^Met ^15,16 (Fig. 1A Construct 1). Previous work has also led to the identification of *sup3–5* as an opal suppressing tRNA^Ser^, which is known to suppress the adenine auxotrophic mutant *ade6–704* (Fig. 1A Construct 2)^17^,^18^. We designed two DNA constructs in which the tRNA^Met^ was replaced with tRNA^Tyr^ from *B. stearothermophillus* (Fig. 1A Construct 3) or *E. coli* (Fig. 1A Construct 4). All DNA constructs described in Fig. 1A constructs 2, 3, and 4 also carried the marker gene *his5*, into which an amber codon had been inserted in place of the codon for Tyr at position 63. The three DNA constructs were introduced into an *ade6–704 his5Δ* strain also expressing *E. coli* TyrRS (Fig. 1B Strain 1) and selected for growth on medium lacking adenine. Transformants thus obtained were tested for their ability to grow on medium lacking histidine. We found that cells expressing *B. sterothermophillus* or *E. coli* tRNA^Tyr^, but not strains expressing *S. pombe* tRNA^Met^ were capable of growth on medium lacking histidine (Fig. 1C). The suppression efficiency for the heterologous pair, *B. stearothermophillus* tRNA^Tyr^ and *E. coli* TyrRS was significantly lower than the suppression by *E. coli* tRNA^Tyr^ and *E. coli* TyrRS , unlike in mammalian cells in which *B. stearothermophillus* tRNA^Tyr^ was better than *E. coli* tRNA^Tyr^ in effecting suppression^12^,^19^,^20^. These observations established that the bacterial tRNA^Tyr^-TyrRS functioned as an orthogonal pair in fission yeast where they functioned as an amber suppressor. We chose to work with *E. coli* tRNA^Tyr^ in the rest of the study.

To incorporate unnatural amino acids through the expanded genetic code, we employed the mutated *E. coli* TyrRS designated as *E. coli* AzFRS, capable of charging *E. coli* tRNA^Tyr^ with unnatural amino acid azido-phenylalanine (AzF)^9^,^10^. In this experiment, the construct (Fig. 1A Construct 4) carrying the marker for yeast transformation (*sup3–5*) and expressing *E. coli* tRNA^Tyr^ carrying CUA in the anti-codon position was introduced into *ade6–704 his5Δ* fission yeast strains expressing either *E. coli* TyrRS (Fig. 1B Strain 1) or *E. coli* AzFRS (Fig. 1B Strain 2). The construct also carried a *his5* selectable marker into which a UAG stop codon was introduced. Note that all transformants grew on medium lacking adenine, suggesting the existence of plasmids in the strains (Fig. 1D). The suppression of the UAG codon in *his5*, and the resulting growth on the medium lacking histidine, was observed when cells expressed *E. coli* tRNA^Tyr^ and *E. coli* TyrRS, but not in cells carrying the *E. coli* AzFRS and *E. coli* tRNA^Tyr^ (Fig. 1D). Importantly, cells expressing *E. coli* AzFRS and *E. coli* tRNA^Tyr^ were able to grow only on medium lacking histidine but supplemented with AzF, confirming that AzF was genetically incorporated in a mechanism in which the UAG stop codon was read by the translation machinery as a codon for AzF (Fig. 1D).

### Optimization of suppression of *E. coli* tyrosine tRNA pair in fission yeast His5p

**Figure 2:**
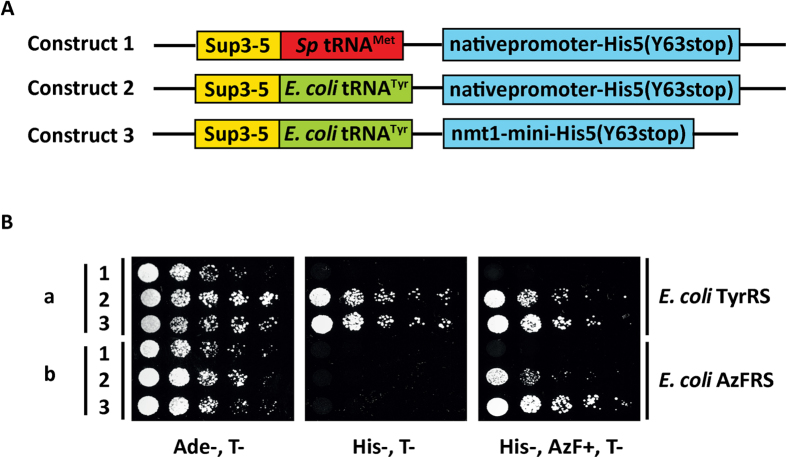
Optimization of the suppression of *E. coli* tRNA^Tyr^/*E. coli* TyrRS pair in *S. pombe*. (**A**) Schematic representation of the constructs. Construct 1: expressing *S. pombe* methionine tRNA (*Sp* tRNA^Met^) and *sup3–5*. His5p, the selectable marker, is expressed under its endogenous promoter. Construct 2: expressing *E. coli* tyrosine tRNA carrying CUA in the anti-codon loop (*E. coli* tRNA^Tyr^) and *sup3–5*. His5p, the selectable marker, is expressed under its endogenous promoter. Construct 3: expressing *E. coli* tyrosine tRNA carrying CUA in the anti-codon loop (*E. coli* tRNA^Tyr^) and *sup3–5*. His5p, the selectable marker, is expressed under *S. pombe nmt1-mini* promoter. (**B**) Spot assay on plates: Constructs 1, 2 and 3 above were introduced to MBY8570 expressing wild type *E. coli* TyrRS and MBY8487 expressing *E. coli* AzFRS. The transformants were selected on Ade- T-, His- T- and His- AzF+ T- media to test the orthogonality, specificity and efficiency of amber suppression. Rows a1–a3: MBY8570 carrying construct 1 or 2 or 3. Rows b1–b3: MBY8487 carrying construct 1 or 2 or 3. Please refer to Supplemental Table S1 for the strain number. T- indicates that cells were placed on agar plates lacking thiamine, which suppresses the *nmt1* promoter.

Effective application of unnatural amino acids to study proteins functions in fission yeast cells is often challenging due to inefficient non-sense suppression and unnatural amino acid incorporation. Since an increase in the expression of the desired mRNA using a strong promoter led to a more robust suppression in budding yeast, we tested if this effect could be recapitulated in fission yeast^21^,^22^. In this experiment, we designed one new construct in which the expression of the gene of interest is driven by the strong inducible *nmt1-mini* promoter (Singh *et al*., unpublished observations), which contains 400 bp of nmt1 promoter (Fig. 2A Construct 3). The gene we tested was a *his5* mutant with an amber codon in place of the codon for Tyr63. Both *E. coli* tRNA^Tyr^ and targeted gene *his5* are encoded on a pNO13 plasmid (Fig. 2A). Spot assays showed that all transformants grew on medium lacking adenine (Fig. 2B). Suppression of the UAG stop codon in *his5* and thereby growth on medium lacking histidine was observed when cells expressed *E. coli* tRNA^Tyr^ and *E. coli* TyrRS (Fig. 2B row a2 and a3), and in cells carrying the *E. coli* tRNA^Tyr^ and *E. coli* AzFRS (Fig. 2B row b2 and b3). However, suppression was observed in AzFRS expressing cells only when AzF was supplemented in the medium. As expected, suppression was not observed in cells expressing *E. coli* TyrRS or *E. coli* AzFRS in the absence of *E. coli* tRNA^Tyr^ (Fig. 2B row a1 and b1). Importantly, suppression of the UAG stop codon in *his5* is stronger when *his5* expression was under control of the *nmt1-mini* promoter (Fig. 2B row a3 and b3), compared to the native *his5* promoter (Fig. 2B row a2 and b2). Through these experiments we established that overexpression of the mRNA of interest (carrying the UAG codon) increased the efficiency of growth in medium lacking histidine, potentially due to the increase in the overall level of the suppressible mRNA.

### Visualization of the suppression of *E. coli* tyrosine tRNA pair *in vivo* and *in vitro*

**Figure 3:**
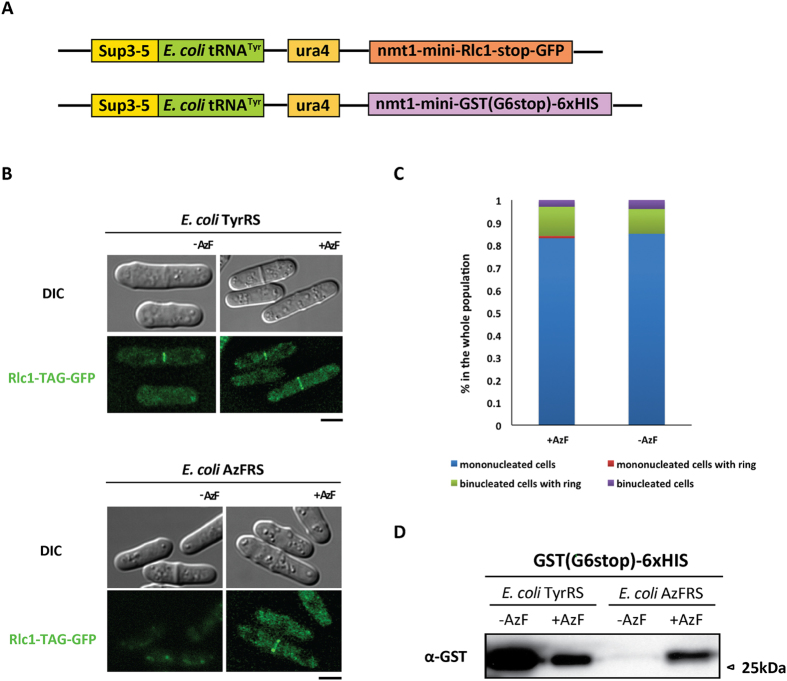
Detection of unnatural amino acid incorporation by Western blot and fluorescence imaging. (**A**) Schematic representation of mutation sites in Rlc1p-GFP and Glutathion-S-transferase (GST). (**B**) Top: *S. pomb*e cells expressing *E. coli* TyrRS and Rlc1p-TAG-GFP from a plasmid (MBY 8965) were grown at 30 °C in the presence or absence of 1 mM azido-phenylalanine to exponential growth phase and live cells were imaged using Andor Spining Disk. 5 μm. Bottom: *S. pombe* cells expressing *E. coli* AzFRS and Rlc1p-TAG-GFP from a plasmid (MBY 8966) were grown at 30 °C in the presence or absence of 1 Mm azido-phenylalanine to exponential growth phase. Cell were imaged using Andor Spining Disk. 5 μm. Please refer to Supplemental Table S1 for the strain numbers. (**C**) AzF has no adverse effect on cell growth and cell cycle progression. The *E. coli* TyrRS cells expressing Rlc1p-TAG-GFP in the absence or presence of AzF were quantified. The relative proportions of mononucleated cells (shown as blue bars), mononucleated cells with actomyosin ring (shown as red bars), binucleated cells (shown as purple bars), and binucleated cells with actomyosin ring (shown as green bars) were quantitated. The mean relative proportion of each category from three independent experiments is shown (N > 60 for each strain in each experiment). (**D**) The cell extract were prepared from *E. coli* TyrRS strain expressing GST (G6stop)-6xHIS (MBY 10031) in the AzF+ and AzF− medium, respectively. The same as *E. coli* AzFRS strain expressing GST (G6stop)-6xHIS (MBY 10026). In total, all the four samples were purified with Ni-NTA, respectively and detected with α-GST antibodies. Please refer to Supplemental Table S1 for the strain number.

We next investigated the ability of tRNA^Tyr^, TyrRS and AzFRS to suppress amber codon mutations in mRNA encoding a synthetic peptide fused between myosin II light chain (Rlc1p) and GFP and in mRNA encoding Glutathione-S-Transferase (GST) in place of the codon for Gly at position 6 (Fig. 3A).

To investigate suppression of amber stop codon *in vivo*, a DNA molecule in which one amber stop codon TAG was placed between *rlc1* and GFP was introduced into an *ade6–704 his5Δ* strain expressing either TyrRS or AzFRS and GFP fluorescence was monitored. In the strain expressing TyrRS, GFP fluorescence in the actomyosin ring was observed in the presence or in the absence of AzF in the medium. However, in the strain expressing the AzFRS, GFP fluorescence was detected in the ring only when cells were grown on medium containing AzF (Fig. 3B). We ensured that the lack of rings in medium lacking AzF was not due to a defect in entry into mitosis (Fig. 3C), since the proportion of binucleate cells in the strain expressing TyrRS in the absence of AzF was not significantly different from the growth in the presence of AzF.

Next we checked suppression when a UAG codon was introduced into RNA encoding a third reporter protein Glutathione-S-Transferase, GST. Again, suppression was observed in the presence and absence of AzF when TyrRS was expressed. However, full length GST was not detected in the cells expressing AzFRS when cultured in the absence of AzF, but was present when cells were grown in the presence of AzF (Fig. 3D). Presently, we do not understand why the suppression is reduced in the presence of AzF when TyrRS and tRNA^Tyr^ are used. It is possible that AzF and Tyr may compete for a similar binding site on TyrRS, which might affect the incorporation of Tyr in the presence of AzF, notwithstanding the fact that tRNA^Tyr^ can only be charged with tyrosine. Collectively, our studies have shown efficient non-sense suppression within 3 mRNAs and also that the unnatural amino acid AzF can be introduced in a site-specific manner into fission yeast proteins.

### Azido-phenylalanine incorporation in response to an amber stop codon

**Figure 4:**
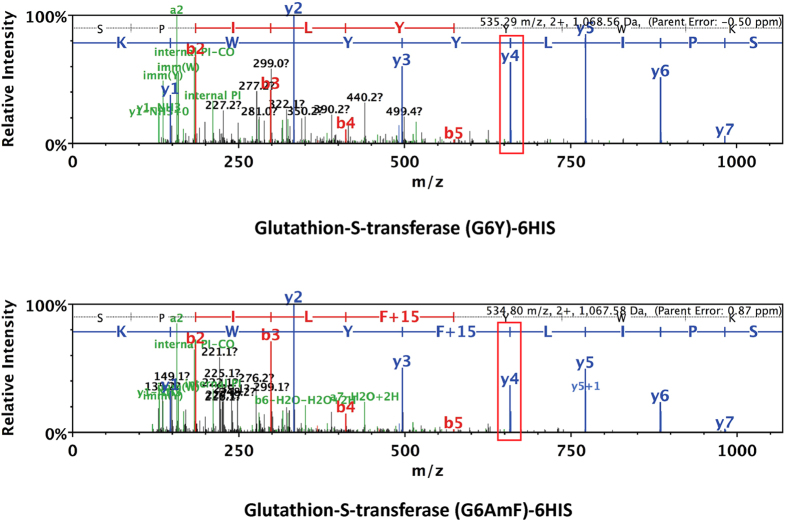
NanoLC-ESI-MS/MS Analysis of Glutathione-S-transferase containing the incorporated amino acids. (**A**) The spectra of tryptic peptide MSPILG*YWK incorporating into tyrosine at glycine 6^th^ (denoted G*) is shown with its expected fragment ion mass y4 (in red square). Parent error:-0.50 ppm. (**B**) The spectra of tryptic peptide MSPILG*YWK incorporating into unnatural amino acid—amino-phenylalanine at glycine 6th (denoted G*) is shown with its expected fragment ion mass y4 (in red square). Parent error:-3.5 ppm.

To further verify site-specific incorporation of AzF into the protein of interest and to determine its occupancy at the amber position (position 6) rather than any other amino acid, the GST protein purified from cells expressing TyrRS, GST (G6Y), and from cells expressing AzFRS, GST (G6AzF), were analyzed by nano LC-ESI-MS/MS after digestion with trypsin. The GST peptide (SPILYYWK) that incorporates tyrosine at position 6, was readily identified by mass spectrometry from cells expressing TyrRS as a 2+ ion of 535.29 *m*/*z* (Fig. 4 top). Curiously, despite the AzF-dependent suppression of the UAG codon in GST protein upon expression of AzFRS, we were unable to detect the expected mass increase of 131 Da from the wild type GST peptide SPILGYWK (Supplemental Fig. 1A) that would represent incorporation of AzF. However, we identified a 2+ ion of 534.80 *m*/*z* that fragmented to match peptide SPILFYWK with the F modified by an additional 15 Da of mass. The position of the F +15 Da modification is supported by y ions 4, 5, 6, 7 (Fig. 4 bottom). It is known that AzF can be reduced to *p*-amino-L-phenylalanine (AmF) during sample preparation^9^,^10^,^23^. We suggest that AzF at position 6 was reduced to AmF, which would be consistent with the molecular weight observed (Supplemental Fig. 1B). Taken together, we conclude that AzF is site specifically incorporated in response to an amber codon into *S. pombe* proteins and is readily converted into AmF.

### Covalent cross-linking in GST upon incorporation of AzF

**Figure 5:**
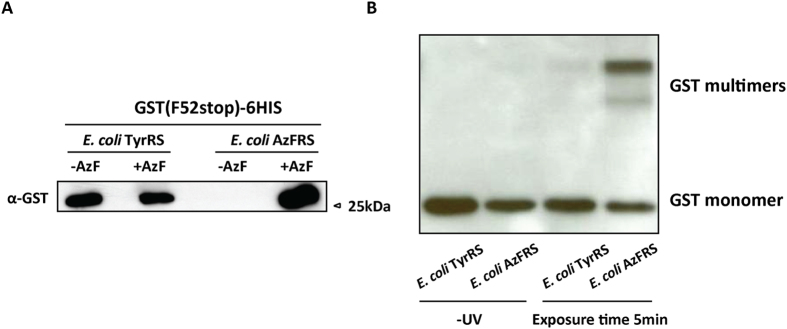
Cross-linking induction with GST (F52AzF)-6HIS. (**A**) *S. pombe* cells expressing *E. coli* TyrRS (MBY 8570) or *E. coli* AzFRS (MBY 8487), both were transformed with *E. coli* tRNA^Tyr^ and GST with a chain terminating UAG stop codon at amino acid position 52 (a site known to be involved in GST dimerization). GST was purified following non-sense UAG suppression. Purified GST from all four samples were analyzed by GST antibody. Please refer to Supplemental Table S1 for the strain number. (**B**) The purified GST was exposed to UV light at 254 nm for 5 min and western blot with GST antibodies.

Azido-phenylalanine is a nitrene generating cross-linking amino acid, whose reactive nitrenes can be generated by exposure of the protein with ultraviolet light. Although the bulk of AzF appeared to be converted to AmF, we reasoned that a fraction of AzF might still be present in the mixture and might be able to covalently cross-link with interacting proteins. To develop this system further, we generated GST with a UAG codon in place of the codon for Phe52, a site known to be involved in GST-dimerization^24^. UAG codon suppression was achieved upon expression of TyrRS and AzFRS, in which case suppression required the presence of AzF in the medium (Fig. 5A). Upon exposure to UV, GST carrying AzF at Phe52, but not GST carrying tyrosine at Phe52, was converted into covalently linked multimers that ran slower on SDS-PAGE (Fig. 5B). These experiments established that despite the conversion of the majority of AzF to AmF a fraction of GST may contain AzF which aids in UV cross-linking.

## Discussion

In this study, we have developed the tools to introduce the unnatural amino acid AzF in a site-specific manner into *Schizosaccharomyces pombe* proteins. We have achieved this by the generation of *S. pombe* strains expressing orthogonal pairs of bacterial tRNA (that base pair with the UAG non-sense codon) and aminoacyl-tRNA synthetases (for natural or unnatural amino acids). Using this approach we have succeeded in introducing tyrosine and the nitrene-generating unnatural amino acid, azido-phenyl alanine (AzF) into three proteins (GST, His5p, and a synthetic linker peptide). We did not observe any major deleterious phenotypic effects in cells carrying non-sense suppressor tRNA and aaRSs. Thus non-sense suppression based on introduction of unnatural amino acids can be used to study fission yeast protein structure and function. This work has also established that the *E. coli* and *B. stearothemophilus* tRNA^Tyr^ and *E. coli* TyrRS function as an orthogonal pair in fission yeast.

Although non-sense suppression was only observed in the presence of all three factors, 1. tRNA^Tyr^, 2. AzFRS, and 3. AzF in the growth medium, we were unable to detect AzF in mass spectrometric analysis. Rather we found a peak consistent with conversion of AzF to amino-phenylalanine (as described in previous work)^9^,^10^,^23^. The conversion of AzF to AmF reduced the utility of the current system we have developed. Additional work is required to prevent reduction of AzF to AmF, so that the system can be used in protein-protein interaction studies. We have detected covalent cross-links between GST molecules, suggesting that despite our inability to detect an AzF peak in mass spectrometry, a fraction of GST should contain unreduced *bona fide* AzF.

In western blot analysis, we found that the amount of GST was less in non-sense suppressed strains compared to the level of GST expressed from mRNA that did not have an internal non-sense codon (data not shown). This is consistent with previous work that non-sense suppression is an inefficient process^12^. In addition, non-sense mediated decay of mRNA might also affect transcript abundance, as has been shown in *C. elegans* and *S. cerevisiae*^11^,^25^. Consistently, an increase in non-sense suppression was observed in *S. cerevisiae*, strains defective in the NMD pathway^26^. Development of such strains should increase the non-suppression efficiency in fission yeast.

Site-specific incorporation of AzF has been achieved in the budding yeasts *S. cerevisiae* and *C. albicans*. However, the biology of *S. pombe* allows for the investigation of a number of cell biological questions (such as cytokinesis and RNA interference) that are not easily tackled in budding yeasts. Our work on incorporation of site-specific incorporation of AzF, thus fills an important gap and opens up a new avenue to study fission yeast protein function. Further refinement of this method is necessary to allow full utilization of this approach to investigate protein function.

## Methods

### Media and growth conditions

Cells were grown and maintained in the yeast extract medium (YES) or Edinburgh minimal medium with appropriate supplements, as described in Moreno *et al*. (1991)^27^. YPD medium containing 1% yeast extract (Gibco-BRL), 2% peptone (Gibco-BRL) and 2% glucose was mainly used for mating and sporulation. All experiments were done at 30 °C which is the optimal growth temperature for tRNA^Tyr^-TyrRS expressing fission yeast strains, unless indicated otherwise. To suppress the stop codon in *rlc1*-TAG-*gfp* which was expressed under *nmt1* promoter control, cells were grown in thiamine containing medium (repressing condition) overnight, followed by three washes with medium lacking thiamine and growth for another 6 h in medium lacking thiamine but supplemented with unnatural amino acid before imaging.

### Plasmid construction

All the polymerase chain reactions are performed on a T100 Thermal Cycler from BIO-RAD. Inverse PCR based plasmid construction method was used to generate tRNA^Tyr^ expression constructs as well as mutations on the *gst* and *rlc1-gfp*. For inverse PCR, two primers were designed to introduce insertion fragment. Each primer was ~50 bp in length and contained ~25 bp overlap with the plasmid backbone sequence. A standard PCR was performed firstly to generate the insertion fragment. 500 ng of the purified PCR product was used as primer in a 50 μL inverse PCR mixture which contained 30 ng of plasmid template, 5 μL of dNTP, 5 μL of Pfu buffer, 1 μL Pfu and ddH_2_O made up to 50 μL. For site-directed mutagenic PCR, most steps were the same as in the inverse PCR. The only difference was the targeted mutation was designed into the primers and located into the middle of the overlap region of forward primer and reverse primers.

### *S. pombe* Lithium Acetate transformation

To transform plasmids into fission yeast cell, 20 mL overnight culture (a concentration of 0.5–1 × 10^7^ cells/ml (OD_595_ ≈ 0.5)) was harvested at 3,000 rpm for 1 min and washed with 1 mL LiAc/TE buffer (made from 100 mM lithium acetate pH 7.5, 10 mM Tris-HCl and 1 mM EDTA pH 7.5). After suspending cells in 100 μL LiAc/TE buffer, 2 μL SSDNA (sonicated salmon sperm carrier DNA, Stratagene) and 2 μg DNA fragments (linearized) or plasmids were added to the suspended cells and incubated at room temperature for 15 min. Next, 240 μL of PEG/LiAc/TE (40% w/v PEG 4000 in 100 mM LiAc, 10 mM Tris-HCl, 1 mM EDTA) was added to cells followed by gentle mixing. After incubating them at 30 °C for 45–60 min, 43 μL of dimethyl sulfoxide (DMSO) was added to the cells followed by gentle shaking. Cells were heated at 42 °C for 5 min and rinsed with sterile water. Cells were resuspended in 100 μL water and plated on the selective agar plates.

### Western Blot

Cells were lysed by glass bead disruption and eluted with lysis buffer (50 mM Tris-HCl pH 7.4, 150 mM NaCl, 5 mM EDTA, 1 mM PMSF, supplemented with protease inhibitors (Complete EDTA-free; Roche Diagnostics, Basel, Switzerland)). The lysate was clarified by centrifugation at 200 *g*. The supernatant was heated at 100 °C for 5 minutes after the addition of 2× Laemmli sample buffer and dithiothreitol. For western blotting, equal volumes of each sample were loaded on SDS- polyacrylamide gels of appropriate concentration and run at a constant current of 20 mA for 80 min. Note that the maximum voltage was lower than 200 V. After SDS-PAGE, proteins were blotted to PVDF (Polyvinylidene fluoride) membranes at a constant voltage of 90 V for 100 min at 4 °C. Transfer buffer used was 25 mM Tris-HCl pH 8.3, 192 mM glycine, 10% v/v methanol. Membranes were incubated with appropriate concentration of primary antibodies in blocking buffer (5% w/v milk, 0.05% v/v Tween-20 in TBS or PBS) at room temperature for 3 h or 4 °C overnight. After washing the blots thrice with PBST or TBST with gentle agitation, secondary antibodies were added and incubated at room temperature for 1 h. Signals were detected using the ECL approach^28^.

### Glutathione S-transferase purification

Cell extract preparation for the glutathione S-transferase purification was as described for the western blot experiments. The extracts were incubated for 1–2 h at 4 °C with 20 μL of pre-swollen glutathione-sepharose 4B (GE healthcare) beads equilibrated with lysis buffer (150 mM NaCl, 50 mM Tris pH 8.0, 10% Glycerol, complete protease inhibitors and 1.5 μg/mL of PMSF). The supernatants were removed after centrifugation at a speed of 1,000 rpm for 2 min. The beads were washed with PBS containing protease inhibitors twice and lysis buffer containing 0.1% Triton-X100 once. The final elution buffer was 50 mM Tris/HCl, 10 mM reduced Glutathione, pH 8.0. Two fractions were collected.

### His-tag purification

Cell extracts from 300 mL cell for the His purification was prepared as described for the western blot experiments. The extracts were incubated for 1–2 h at 4 °C with 300 μL of pre-washed HisPur Ni-NTA Resin (Thermo Scientific) equilibrated with lysis buffer (150 mM NaCl, 50 mM Tris-Cl pH 8.0, 10% glycerol, complete protease inhibitors and 1.5 μg/mL of PMSF). The supernatants were removed after spinning down at a speed of 2,000 rpm for 2 min. The beads were washed with lysis buffer with 20 mM imidazole thrice. The final elution buffer was lysis buffer contained 500 mM imidazole.

### GST UV Crosslinking

Purified GST proteins containing unnatural amino acid AzF or tyrosine were used in this experiment. The concentration of purified proteins was estimated and diluted to equivalent levels. 20 uL of each sample was loaded on parafilm, which was kept on an icebox. Samples were exposed to UV (254 nm) in UV crosslinker CL-1000 (VWR) for 5 min and collected into a microfuge tube.

### Spinning Disk Microscopy

Spinning disk images were captured by Andor spinning disk. Andor spinning disk using a Nikon ECLIPSE Ti-E microscopy equipped with a Plan Apo Vc 100×/1.40 N.A. Oil objective lens, a Yokogawa CSU-X1 unit spinning disk system, Andor iXon Ultra EMCCD (16 um/pxl) camera with 2× adapter for CSU-X1 and Andor iQ3 software. 405, 488, 561 and 640 nm solid state diode (power 50/100 mW) laser were used for excitation. All images were processed and analyzed by using ImageJ (http://rsb.info.nih.gov/ij/) software. Graphs in this paper were made by Excel.

## Additional Information

**How to cite this article**: Shao, N. *et al*. Site Specific Genetic Incorporation of Azidophenylalanine in *Schizosaccharomyces pombe*. *Sci. Rep*. **5**, 17196; doi: 10.1038/srep17196 (2015).

## Supplementary Material

Supplementary Information

## References

[b1] Johansson L., Gafvelin G. & Arner E. S. Selenocysteine in proteins-properties and biotechnological use. Biochimica et biophysica acta 1726, 1–13, doi: 10.1016/j.bbagen.2005.05.010 (2005). 15967579

[b2] Hao B. . A new UAG-encoded residue in the structure of a methanogen methyltransferase. Science (New York, NY) 296, 1462–1466, doi: 10.1126/science.1069556 (2002). 12029132

[b3] Srinivasan G., James C. M. & Krzycki J. A. Pyrrolysine encoded by UAG in Archaea: charging of a UAG-decoding specialized tRNA. Science (New York, NY) 296, 1459–1462, doi: 10.1126/science.1069588 (2002). 12029131

[b4] Noren C. J., Anthony-Cahill S. J., Griffith M. C. & Schultz P. G. A general method for site-specific incorporation of unnatural amino acids into proteins. Science (New York, NY) 244, 182–188 (1989). 10.1126/science.2649980 2649980

[b5] Furter R. Expansion of the genetic code: site-directed p-fluoro-phenylalanine incorporation in Escherichia coli. Protein science: a publication of the Protein Society 7, 419–426, doi: 10.1002/pro.5560070223 (1998). 9521119 PMC2143905

[b6] Liu D. R. & Schultz P. G. Progress toward the evolution of an organism with an expanded genetic code. Proceedings of the National Academy of Sciences of the United States of America 96, 4780–4785 (1999). 10220370 10.1073/pnas.96.9.4780 PMC21768

[b7] Wang L., Brock A., Herberich B. & Schultz P. G. Expanding the genetic code of Escherichia coli. Science (New York, NY) 292, 498–500, doi: 10.1126/science.1060077 (2001). 11313494

[b8] Liu C. C. & Schultz P. G. Adding new chemistries to the genetic code. Annual Review of Biochemistry 79, 413–444, doi: 10.1146/annurev.biochem.052308.105824 (2010). 20307192

[b9] Chin J. W. . An expanded eukaryotic genetic code. Science (New York, NY) 301, 964–967, doi: 10.1126/science.1084772 (2003). 12920298

[b10] Palzer S. . An expanded genetic code in Candida albicans to study protein-protein interactions *in vivo*. Eukaryotic Cell 12, 816–827, doi: 10.1128/ec.00075-13 (2013). 23543672 PMC3675983

[b11] Parrish A. R. . Expanding the genetic code of Caenorhabditis elegans using bacterial aminoacyl-tRNA synthetase/tRNA pairs. ACS Chemical Biology 7, 1292–1302, doi: 10.1021/cb200542j (2012). 22554080 PMC3401359

[b12] Sakamoto K. . Site-specific incorporation of an unnatural amino acid into proteins in mammalian cells. Nucleic Acids Research 30, 4692–4699 (2002). 12409460 10.1093/nar/gkf589 PMC135798

[b13] Liu W., Brock A., Chen S., Chen S. & Schultz P. G. Genetic incorporation of unnatural amino acids into proteins in mammalian cells. Nature Methods 4, 239–244, doi: 10.1038/nmeth1016 (2007). 17322890

[b14] Bedouelle H. Recognition of tRNA(Tyr) by tyrosyl-tRNA synthetase. Biochimie 72, 589–598 (1990). 2126463 10.1016/0300-9084(90)90122-w

[b15] Otter C. A. & Straby K. B. Transcription of eukaryotic genes with impaired internal promoters: the use of a yeast tRNA gene as promoter. Journal of Biotechnology 21, 289–293 (1991). 1367696 10.1016/0168-1656(91)90049-2

[b16] Otter C. A., Edqvist J. & Straby K. B. Characterization of transcription and processing from plasmids that use polIII and a yeast tRNA gene as promoter to transcribe promoter-deficient downstream DNA. Biochimica et biophysica acta 1131, 62–68 (1992). 1581361 10.1016/0167-4781(92)90099-l

[b17] Grallert B., Nurse P. & Patterson T. E. A study of integrative transformation in Schizosaccharomyces pombe. Molecular & General Genetics: MGG 238, 26–32 (1993). 8479431 10.1007/BF00279526

[b18] Hottinger H. . The Schizosaccharomyces pombe sup3-i suppressor recognizes ochre, but not amber codons *in vitro* and *in vivo*. The EMBO Journal 3, 423–428 (1984). 6370683 10.1002/j.1460-2075.1984.tb01823.x PMC557361

[b19] Capone J. P., Sedivy J. M., Sharp P. A. & RajBhandary U. L. Introduction of UAG, UAA, and UGA nonsense mutations at a specific site in the Escherichia coli chloramphenicol acetyltransferase gene: use in measurement of amber, ochre, and opal suppression in mammalian cells. Molecular and Cellular Biology 6, 3059–3067 (1986). 3023959 10.1128/mcb.6.9.3059-3067.1986 PMC367040

[b20] Young J. F. . Measurement of suppressor transfer RNA activity. Science (New York, NY) 221, 873–875 (1983). 10.1126/science.6308765 6308765

[b21] Chen S., Schultz P. G. & Brock A. An improved system for the generation and analysis of mutant proteins containing unnatural amino acids in Saccharomyces cerevisiae. Journal of Molecular Biology 371, 112–122, doi: 10.1016/j.jmb.2007.05.017 (2007). 17560600

[b22] Partow S., Siewers V., Bjorn S., Nielsen J. & Maury J. Characterization of different promoters for designing a new expression vector in Saccharomyces cerevisiae. Yeast (Chichester, England) 27, 955–964, doi: 10.1002/yea.1806 (2010). 20625983

[b23] Nehring S., Budisa N. & Wiltschi B. Performance analysis of orthogonal pairs designed for an expanded eukaryotic genetic code. PloS one 7, e31992, doi: 10.1371/journal.pone.0031992 (2012). 22493661 PMC3320878

[b24] Dirr H., Reinemer P. & Huber R. X-ray crystal structures of cytosolic glutathione S-transferases. Implications for protein architecture, substrate recognition and catalytic function. European Journal of Biochemistry/FEBS 220, 645–661 (1994). 8143720 10.1111/j.1432-1033.1994.tb18666.x

[b25] Wang Q. & Wang L. New methods enabling efficient incorporation of unnatural amino acids in yeast. Journal of the American Chemical Society 130, 6066–6067, doi: 10.1021/ja800894n (2008). 18426210

[b26] Guan Q. . Impact of nonsense-mediated mRNA decay on the global expression profile of budding yeast. PLoS Genetics 2, e203, doi: 10.1371/journal.pgen.0020203 (2006). 17166056 PMC1657058

[b27] Moreno S., Klar A. & Nurse P. Molecular genetic analysis of fission yeast Schizosaccharomyces pombe. Methods in Enzymology 194, 795–823 (1991). 2005825 10.1016/0076-6879(91)94059-l

[b28] Madamanchi N. R. & Runge M. S. Western blotting. Methods in Molecular Medicine 51, 245–256, doi: 10.1385/1-59259-087-x:245 (2001). 21331721

