# Corrigendum: Site Specific Genetic Incorporation of Azidophenylalanine in *Schizosaccharomyces pombe*

**DOI:** 10.1038/srep21354

**Published:** 2016-02-22

**Authors:** Nan Shao, N. Sadananda Singh, Susan E. Slade, Alexandra M. E. Jones, Mohan K. Balasubramanian

Scientific Reports 5: Article number: 1719610.1038/srep17196; published online: 11242015; updated: 02222016

This Article contains errors in the Acknowledgements section:

“We express our sincere gratitude to the WPH Proteomics Research Technology Platform for the contribution to the mass spectrometry analysis. In addition, we would like to express our thanks to MKB laboratory members for discussion. This work was supported by research funds from National University of Singapore, Wellcome Trust (WT101885MA), The Royal Society, Temasek Life Sciences Laboratory and University of Warwick.”

should read:

“We express our sincere gratitude to the WPH Proteomics Research Technology Platform for the contribution to the mass spectrometry analysis. In addition, we would like to express our thanks to Prof. Thomas Sakmar (Rockefeller University) for providing us the tRNA-synthetase pair. In the end, we would like to thank all the MKB laboratory members for discussion. This work was supported by research funds from National University of Singapore, Wellcome Trust (WT101885MA), The Royal Society, Temasek Life Sciences Laboratory and University of Warwick.”

