# Supplementary material for: Site Specific Genetic Incorporation of Azidophenylalanine in Schizosaccharomyces pombe
Source: Sci Rep. 2015 Nov 24;5:17196. doi: 10.1038/srep17196 (PMC4657001; doi:10.1038/srep17196)
Supplement: Supplementary Information [file srep17196-s1.pdf]

Supplemental Information

Site Specific Genetic Incorporation of Azidophenylalanine in *Schizosaccharomyces pombe*

Nan Shao[1 2 3], N. Sadananda Singh[3], Susan E. Slade[4], Alexandra M.E. Jones[4], *Mohan K. Balasubramanian[1 2 3 5]

[1] Division of Biomedical Cell Biology, Warwick Medical School, University of Warwick, Coventry, UK CV4 7AL

[2] Department of Biological Sciences, National University of Singapore, Singapore 117543

[3] Temasek Life Sciences Laboratory, National University of Singapore, 1 Research Link, Singapore 117604

[4] School of Life Sciences, University of Warwick, Coventry, UK CV4 7AL

[5] Mechanobiology Institute, 5A Engineering Drive 1, National University of Singapore, Singapore 117411

*Corresponding author

Correspondence to Mohan K. Balasubramanian: Email: M.K.Balasubramanian@warwick.ac.uk

Supplemental Inventory

Supplemental Figure Schematic representation of the conversion between azido-phenylalanine and amino-phenylalanine

Supplemental Table S1. List of strains used in this study

Supplemental Table S2. List of constructs generated in this study

Supplemental Methods

Trichloroacetic Acid (TCA) Precipitation and Filter Aided Sample Preparation (FASP)

Gel processing, enzymatic digestion and peptide extraction

nanoLC-ESI-MS/MS Analysis

MS Data Analysis

Supplemental Figure

A

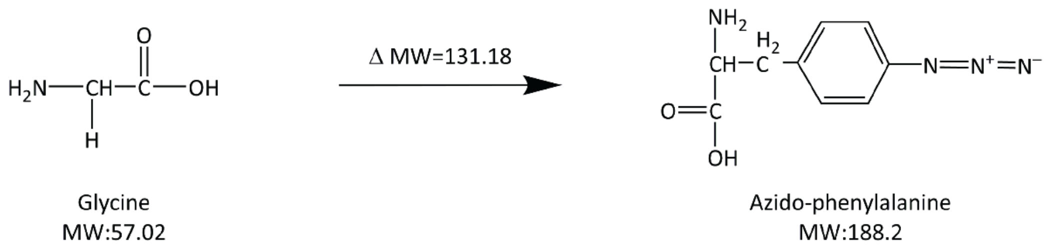

B

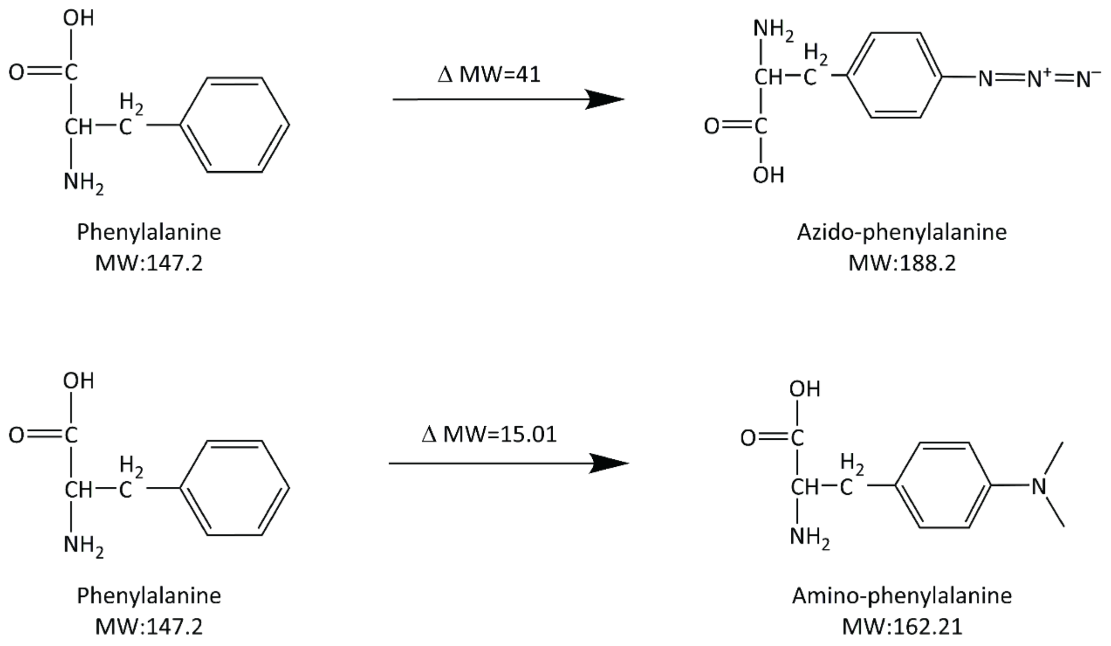

Supplemental Figure Schematic representation of the conversion between azido-phenylalanine and amino-phenylalanine

(A) The mass difference between glycine and azido-phenylalanine is 131.18 kDa.

(B) The mass difference between phenylalanine and azido-phenylalanine is 41 kDa; The mass difference between phenylalanine and amino-phenylalanine is 15.01 kDa

Supplemental Table S1: *S. pombe* strains used in this study

Strain No.	Genotype	Source
MBY7903	ade6-704 ura4-294 leu1-32 his5DEL	This study
MBY8487	act1 promoter -azpRS::leu, ade6-704 ura4-294 leu1-32 his5DEL	This study
MBY8570	act1 promoter -EcoYRS(WT)-FLAG::leu1+ ade6-704 ura4-294 his5DEL	This study
MBY8654	ade6-704 ura4-294 leu1-32 his5DEL, pNO13T-his5TAG-sup3.5-met	This study
MBY8655	ade6-704 ura4-294 leu1-32 his5DEL , pNO13T-his5TAG-sup3.5-EcoYtRNA	This study
MBY8744	act1 promoter -EcoYRS(WT)-FLAG::leu1+ ade6-704 ura4-294 his5DEL, pNO13-sup3.5-ecoYtRNA-nmt1-mini-his5TAG-6xhis-tag	This study
MBY8745	act1 promoter -EcoYRS(WT)-FLAG::leu1+ ade6-704 ura4-294 his5DEL, pNO13-sup3.5-ecoYtRNA-nmt1-mini-GST-52-TAG	This study
MBY8746	act1 promoter -azpRS::leu, pNO13T-his5TAG-sup3.5-EcoYtRNA	This study
MBY8747	act1 promoter -azpRS::leu, pNO13-sup3.5-ecoYtRNA-nmt1-mini-his5TAG-6xhis-tag	This study
MBY8748	act1 promoter -azpRS::leu, pNO13-sup3.5-ecoYtRNA-nmt1-mini-GST-52-TAG	This study
MBY8897	act1 promoter -EcoYRS(WT)-FLAG::leu1+, pNO13-sup3.5-ecoYtRNA-ura4-nmt1-mini-GST-52-TAG-6xhis-tag	This study
MBY8898	act1 promoter -azpRS::leu, pNO13-sup3.5-ecoYtRNA-ura4-nmt1-mini-GST-52-TAG-6xhis-tag	This study
MBY8965	act1 promoter -EcoYRS(WT)-FLAG::leu1+, pNO13-sup3.5-ecoYtRNA-ura4- nmt1-mini-rlc1-TAG-gfp	This study
MBY8966	act1 promoter -azpRS::leu+, pNO13-sup3.5-ecoYtRNA ura4- -nmt1-mini-rlc1-TAG-gfp	This study
MBY10026	act1 promoter -azpRS::leu, ade6-704, pNO13-sup3.5-ecoYtRNA-ura4-nmt1-mini-GST(Glycine 6TAG)-6xhis	This study
MBY10031	act1 promoter -EcoYRS(WT)-FLAG::leu1+, ade6-704, pNO13-sup3.5-ecoYtRNA-ura4-nmt1-mini-GST(Glycine6TAG)-6xhis	This study
MBY10104	act1 promoter -EcoYRS(WT)-FLAG::leu1+, pNO13T-his5TAG-sup3.5-met	This study
MBY10105	act1 promoter -EcoYRS(WT)-FLAG::leu1+, pNO13T-his5TAG-sup3.5-EcoYtRNA	This study
MBY10106	act1 promoter -EcoYRS(WT)-FLAG::leu1+, pNO13-sup3.5-EcoYtRNA-nmt1-mini-his5TAG	This study

MBY10107	act1 promoter -azpRS::leu, pNO13T-his5TAG-sup3.5-met	This study
MBY10108	act1 promoter -azpRS::leu, pNO13T-his5TAG-sup3.5-EcoYtRNA	This study
MBY10109	act1 promoter -azpRS::leu, pNO13-sup3.5-ecoYtRNA-nmt1-mini-his5TAG	This study
MBY10110	act1 promoter -benzoylRS::leu, ade6-704, ura4-294, his5DEL	This study
MBY10111	act1 promoter -benzoylRS::leu, ade6-704, ura4-294, his5DEL , pNO13T-his5TAG-sup3.5-EcoYtRNA	This study
MBY10112	act1 promoter -benzoylRS::leu, ade6-704, ura4-294, his5DEL , pNO13-sup3.5-ecoYtRNA-nmt1-mini-his5TAG	This study
MBY10113	act1 promoter -benzoylRS::leu, ade6-704, ura4-294, his5DEL , pNO13-sup3.5-ecoYtRNA-nmt1-mini-his5TAG	This study

Supplemental Table S2: List of all the constructs generated in this study.

pTMB No.	Vector
pTMB 75	pNO13T-spleu6flanks-bstYtRNA
pTMB 76	pNO13T-his5TAG-spleu6flanks-bstYtRNA
pTMB 77	pNO13T-his5TAG-sup3.5-bstYtRNA
pTMB 78	pNO13-ser3-TAG
pTMB 79	pJK148-act1promoter-EcoYRS(WT) with FLAG-tag at C-terminus
pTMB 80	pNO13T-his5TAG-sup3.5-met
pTMB 81	pNO13T-his5TAG-sup3.5-met-spleuflanks-BstYtRNA
pTMB 82	pNO13T-his5TAG-sup3.5-EcoYtRNA
pTMB 83	pNO13T-his5TAG-sup3.5-met-spleuflanks-EcoYtRNA
pTMB 84	pNO13-sup3.5-EcoYtRNA
pTMB 85	pNO13-ser3-EcoYtRNA
pTMB 86	pNO13-sup3.5-ecoYtRNA-nmt1-mini-his5TAG
pTMB 87	pNO13-sup3.5-ecoYtRNA-nmt1-mini-GST-52-TAG
pTMB 89	pNO13-sup3.5-ecoYtRNA-nmt1-mini-his5TAG-6xhis-tag
pTMB 103	pNO13-sup3.5-ecoYtRNA-ura4-nmt1-mini-GST-52-TAG-6xhis-tag
pTMB 109	pJK148-act1promoter-EcoYRS-FLAG
pTMB 115	pJK148-act1 promoter-azidoRS
pTMB 146	pNO13-sup3.5-ecoYtRNA-ura4-nmt1-mini-rlc1-TAG-gfp
pTMB 147	pNO13-sup3.5-ecoYtRNA-ura4- nmt1-mini-GST(G6amb)-6xhis
pTMB 176	pJK148-act1 promoter-benzyolRS

Supplemental Methods

Trichloroacetic Acid (TCA) Precipitation and Filter Aided Sample Preparation (FASP)

750 µL cold sterilized water was added to 50 µL purified protein. After the addition of 150 µL 1.85 M NaOH, the mixture was vortexed several seconds. 150 µL cold trichloroacetic acid was added into the mixture following by placing on ice 10 min. Then the whole mixture was spun down at a speed of 14,000 rpf around 25 min. The white protein pellet could be observed in the bottom of Eppendorf tube after removing the supernatant.

TCA precipitated sample were suspended in 20 µL lysis buffer (4 % (w/v) SDS, 100 mM Tris/HCl pH 7.6) and kept at 95 °C for 5min. Before starting sample processing, the lysate were clarified by centrifugation at 16,000 *g* for 5 min. The supernatant was transferred to 10 kDa cut-off centrifugal filter (Amicon). The protein extract were mixed up with 400 µL of UA (8M urea (Sigma, U5128) in 0.1 M Tris/Hcl pH 8.8) in the filter unit and centrifuged at a speed of 14,000 *g* for 25 min. This step was repeated one more time and followed by the addition of 100 µL IAA solution (38.5 mg iodoacetamide in UA buffer). The sample was mixed at 600 rpm in thermos-mixer for 1 min and incubated without mixing for 5 min at room temperature. The centrifugal filter unit was spun down at 14,000 *g* for 30 min. After removing the flow-through, 400 µL of UB (8M urea (Sigma, U5128) in 0.1 M Tris/HCl pH 8.0) was added to the filter unit and centrifuged at 14,000 *g* for 40 min. This step was repeated twice. 120 µL ABC (0.05 M NH_4HCO_3 in water) with trypsin was added into the filter unit and incubated at 37 °C overnight. After trypsin digestion, the filter unit was transferred to a new tube and centrifuged at 14,000 *g* 20 min. 50 µL 0.5 M NaCl was added into filter unit, which was spun down at a speed of 14,000 *g* for 20 min. The flow-through was collected and acidified with 0.05 % CF_3COOH.

Gel processing, enzymatic digestion and peptide extraction

All processing of the gel plugs was performed by a MassPrep robotic protein handling system (Micromass, Manchester, UK) using the manufacturer's protocol, described briefly below.

Gel plug/s were destained twice using 50 % acetonitrile in 50 mM ammonium bicarbonate, rinsed with acetonitrile then allowed to air dry for 10 minutes, reduced with 10 mM dithiothreitol in 100 mM ammonium bicarbonate for 30 minutes followed by alkylation with 55 mM iodoacetamide in 100 mM ammonium bicarbonate. The gel plugs were then rinsed with acetonitrile, 100 mM ammonium bicarbonate followed by acetonitrile for a further 3 times. A 25 µL aliquot of 6 ngμL^{-1} trypsin was added to each sample and allowed to incubate at 37 ^{o}C for 4.5 h. The resulting peptides were initially extracted using 30 µL of an aqueous solution containing 2 % acetonitrile and 1 % formic acid. A second extraction using 15 µL of an aqueous solution containing 51 % acetonitrile and 0.5 % formic acid was then performed and combined with the first extraction.

nanoLC-ESI-MS/MS Analysis of GST (G6Y)-6His and GST (G6AzF)-6His

Reversed phase chromatography was used to separate tryptic peptides prior to mass spectrometric analysis. Two columns were utilised, an Acclaim PepMap µ-precolumn cartridge 300 µm i.d. x 5 mm 5 µm 100 Å and an Acclaim PepMap RSLC 75 µm x 25 cm 2 µm 100 Å (Thermo Scientific, San Jose, USA). The columns were installed on an Ultimate 3000 RSLCnano system (Dionex, Sunnyvale, USA). Mobile phase buffer A was composed of 0.1 % aqueous

formic acid and mobile phase B was composed of 80% acetonitrile containing 0.1 % formic acid. Samples were loaded onto the µ-precolumn equilibrated in 2 % aqueous acetonitrile containing 0.1 % trifluoroacetic acid for 8 min at 10 µL min^{-1} after which peptides were eluted onto the analytical column at 300 nL min^{-1} by increasing the mobile phase B concentration from 3 % B to 35 % over 35 min then to 90 % B and held over 4 min and re-equilibrated at 3 % B.

Eluting peptides were converted to gas-phase ions by means of electrospray ionisation and analysed on a Thermo Orbitrap Fusion, (Thermo Scientific, San Jose, USA). Survey scans of peptide precursors from 350 to 1500 *m*/*z* were performed at 120K resolution (at 200 *m*/*z*) with a 4×10^5 ion count target. Tandem MS was performed by isolation at 1.6 Th using the quadrupole, HCD fragmentation with normalized collision energy of 35, and rapid scan MS analysis in the ion trap. The MS^2 ion count target was set to 10^4 and the max injection time was 200 ms. Precursors with charge state 2–7 were selected and sampled for MS^2. The dynamic exclusion duration was set to 45 s with a 10 ppm tolerance around the selected precursor and its isotopes. Monoisotopic precursor selection was turned on. The instrument was run in top speed mode with 2 s cycles.

MS Data Analysis

The raw data was processed using MSConvert in ProteoWizard Toolkit (version 3.0.5759)[1]. MS^2 spectra were searched with Mascot engine (Matrix Science, version 2.5.0) against a database of 5,268 sequences derived from PomBase v2.23, a comprehensive database for fission yeast *Schizosaccharomyces pombe* (http://www.pombase.org) appended with GST sequence and the common Repository of Adventitious Proteins Database (http://www.thegpm.org/cRAP/index.html). Database search parameters assumed digestion enzyme was trypsin, up to two missed cleavage sites were permitted with carbamidomethylation of cysteine as a fixed modification, amino-, azido-phenylalanine and oxidation of methionine were specified variable modifications. Precursor mass tolerance was 20 ppm and product ions were searched at 0.8 Da tolerances.
